# Polyneuropathy, organomegaly, endocrinopathy, monoclonal protein, skin changes (POEMS) syndrome complicated by ischemic stroke: A case report

**Published:** 2018-04-04

**Authors:** Reza Boostani, Zahra Baghestani

**Affiliations:** Department of Neurology, School of Medicine, Mashhad University of Medical Sciences, Mashhad, Iran

**Keywords:** POEMS Syndrome, Brain Ischemia, Vascular Endothelial Growth Factor

A 28-year-old man patient presented with a history of progressive lower limbs weakness followed by bilateral weakness of upper limbs as well as pain in soles for the last two years. He had also developed distal paresthesia involving upper and lower limbs. There was no record of urinary problems. Moreover, there was no history of flu-like or gastrointestinal symptoms at the onset. The only prominent symptoms observed were significant weight loss (about 30 kg), and intense sweating. Family history was unremarkable.

On detailed neurological examination, patient was found to have decreased tone in both upper and lower limbs with absent deep tendon reflexes. Stocking-glove pattern for loss of vibration and pinprick sensation was established. Romberg sign was present and gait was mildly ataxic. No cerebellar signs and symptoms were detected. Neurological examination supported evidence of chronic polyneuropathy. The only abnormal finding from general examination was bilateral gynecomastia.

Results from electrodiagnostic studies were compatible with chronic motor-sensory polyradiculoneuropathy with predominantly demyelinating features and severe degree of secondary axonal loss. Cerebrospinal fluid (CSF) analysis showed normal pattern, except for mildly elevated protein levels. Bone marrow aspiration and biopsy revealed normocellular marrow with 2% plasma cells. Serum protein electrophoresis was normal but immunofixation electrophoresis (IFE) indicated abnormal IgA lambda type band. Endocrine evaluation including thyroid-stimulating hormone (TSH), cortisol, luteinizing hormone (LH), and follicle-stimulating hormone (FSH) were within normal range. The skeletal survey was also normal. Thoracic, abdominal, and pelvic computed tomography (CT) scans showed several discrete axillary, para-aortic, and pelvic lymphadenopathies [maximum source to axis distance (SAD): 12 mm]. Vascular endothelial growth factor (VEGF) serum level was significantly elevated (984.5 pg/ml). 

This constellation of findings including polyneuropathy, monoclonal gammopathy, elevated VEGF, lymphadenopathy, significant weight loss, and hyperhidrosis as well as gynecomastia led to the diagnosis of polyneuropathy, organomegaly, endocrinopathy, monoclonal protein and skin changes (POEMS) syndrome. The patient was started on melphalan and prednisolone. 

After two months, the patient developed new symptoms including right hemiparesis, right central-type facial palsy, and dysphasia with a sub-acute onset. Brain magnetic resonance imaging (MRI) showed evidences of combined left internal borderzone and territorial infarction (Figure 1). Further investigation including transthoracic echocardiography and evaluation of hypercoagulable states did not demonstrate any disorder. Finally, the patient was diagnosed with an ischemic stroke, secondary to underlying POEMS syndrome.

**Figure 1:**
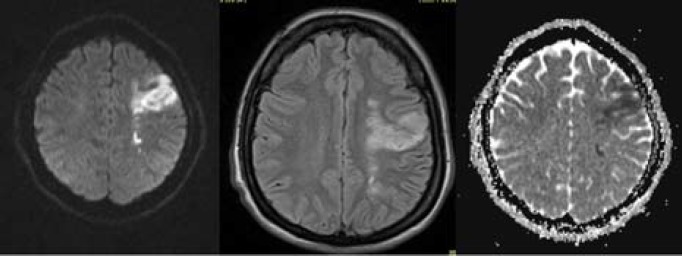
Left internal borderzone and middle cerebral artery (MCA) infarction in magnetic resonance imaging (MRI) study

POEMS syndrome was first introduced by Bardwick, et al. in 1980. This acronym stands for polyneuropathy, organomegaly, endocrinopathy, monoclonal gammopathy and skin changes. Coronary artery disease and ischemia of lower limbs are among rare presentations of POEMS syndrome.^1^^,^^2^ Ischemic stroke has been rarely reported in association with POEMS syndrome as well. To the best of our knowledge, this is the first case with POEMS syndrome, who suffered an ischemic stroke more prominently in left hemispheric internal borderzone, and left middle cerebral artery (MCA) territory, reported in Iran. Results from further investigation such as cardio-embolic etiology and hypercoagulabe states were all normal. 

The findings of a study involving three patients with POEMS syndrome, who experienced an episode of ischemic stroke, showed that stroke was more common in borderzone or watershed areas. In this study, elevated fibrinogen levels were also detected in all three patients.^3^ However, the correlation between elevated fibrinogen levels and ischemic stroke should be confirmed by future studies. In August 2017, a case of cerebral large vessel vasculitis was reported as an unusual manifestation of POEMS syndrome.^4^

The pathophysiologic mechanism of ischemic stroke in patients with POEMS remains unclear. It seems that factors such as polycytemia, thrombocytosis, and elevated proinflammatory cytokines, such as VEGF, may play a role. Nevertheless, the exact mechanism is still largely unknown.^3^^,^^5^^,^^6^ Therefore, it should be noted that secondary prevention of stroke in patients with POEMS syndrome is highly critical in long-term prognosis of these patients

## Notes:

**How to cite this article:** Boostani R, Baghestani Z. Polyneuropathy, organomegaly, endocrinopathy, monoclonal protein, skin changes (POEMS) syndrome complicated by ischemic stroke: A case report. Iran J Neurol 2018; 17(2): 95-6.
